# Antibodies to PcpA and PhtD protect mice against *Streptococcus pneumoniae* by a macrophage- and complement-dependent mechanism

**DOI:** 10.1080/21645515.2017.1403698

**Published:** 2017-12-14

**Authors:** Lucian Visan, Nicolas Rouleau, Emilie Proust, Loïc Peyrot, Arnaud Donadieu, Martina Ochs

**Affiliations:** Sanofi Pasteur, Research & Non Clinical Safety Department, Marcy l'Etoile, France

**Keywords:** antibody, complement, macrophage, neutrophil, opsonophagocytosis, phagocytosis, *Streptococcus pneumoniae*, vaccination

## Abstract

Currently marketed *Streptococcus pneumoniae* (*Spn*) vaccines, which contain polysaccharide capsular antigens from the most common *Spn* serotypes, have substantially reduced pneumococcal disease rates but have limited coverage. A trivalent pneumococcal protein vaccine containing pneumococcal choline-binding protein A (PcpA), pneumococcal histidine triad protein D (PhtD), and detoxified pneumolysin is being developed to provide broader, cross-serotype protection. Antibodies against detoxified pneumolysin protect against bacterial pneumonia by neutralizing *Spn*-produced pneumolysin, but how anti-PhtD and anti-PcpA antibodies protect against *Spn* has not been established. Here, we used a murine passive protection sepsis model to investigate the mechanism of protection by anti-PhtD and anti-PcpA antibodies. Depleting complement using cobra venom factor eliminated protection by anti-PhtD and anti-PcpA monoclonal antibodies (mAbs). Consistent with a requirement for complement, complement C3 deposition on *Spn in vitro* was enhanced by anti-PhtD and anti-PcpA mAbs and by sera from PhtD- and PcpA-immunized rabbits and humans. Moreover, in the presence of complement, anti-PhtD and anti-PcpA mAbs increased uptake of *Spn* by human granulocytes. Depleting neutrophils using anti-Ly6G mAbs, splenectomy, or a combination of both did not affect passive protection against *Spn*, whereas depleting macrophages using clodronate liposomes eliminated protection. These results suggest anti-PhtD and anti-PcpA antibodies induced by pneumococcal protein vaccines protect against *Spn* by a complement- and macrophage-dependent opsonophagocytosis.

Currently marketed *Streptococcus pneumoniae* (*Spn*) vaccines, which are based on polysaccharide capsular antigens from the most common *Spn* serotypes, have substantially reduced the incidence of pneumococcal disease worldwide.^1^ However, coverage by polysaccharide vaccines may be incomplete due to variations in pneumococcal serotypes between countries or regions.^2^ Moreover, serotype replacement has the potential to eventually render these vaccines less effective.^3–5^

In an effort to provide broader and infection stage-specific protection, pneumococcal protein vaccines (PPrVs) based on conserved immunogenic surface proteins are being developed.^6–9^ Key target proteins include pneumococcal choline-binding protein A (PcpA), pneumococcal histidine triad protein D (PhtD), and pneumolysin, which are conserved across *Spn* serotypes.^10^ Due to pneumolysin's toxicity, a detoxified pneumolysin derivative (PlyD1) is used as the vaccine antigen.^11^ Phase I trials have shown that monovalent PhtD^12^ or PlyD1^13^ vaccines, a bivalent PcpA-PhtD protein vaccine,^14^ and most recently, a trivalent PcpA-PhtD-PlyD1 vaccine^10^ are well tolerated and induce antibodies against their respective protein antigens.

Human and mouse antibodies induced by the PPrVs against PcpA, PhtD, and PlyD1 protect mice against a lethal dose of *Spn* in a passive protection sepsis model.^10,15,16^ Antibodies induced by PlyD1 protect against bacterial pneumonia by neutralizing *Spn*-produced pneumolysin, thereby preventing pneumolysin-induced lung lesions and inflammation.^17^ How anti-PcpA and anti-PhtD antibodies protect against *Spn* is less clear. One possibility is that anti-PcpA and anti-PhtD antibodies promote opsonophagocytosis, an important defense mechanism against *Spn*.^18,19^

In mice, antibody-mediated complement deposition on pneumococci initiates opsonophagocytosis and correlates with passive protection against *Spn*.^20^ To determine whether complement plays a role in protection by anti-PcpA and anti-PhtD antibodies, we examined how depleting complement affects passive protection in a CBA/CaHN-Btk^xid^/J (CBA/N) mouse lethal sepsis model. This mouse strain is unable to produce antibodies against pneumococcal polysaccharides and is therefore highly susceptible to *Spn* infection.^21,22^ Mice were injected with cobra venom factor to deplete complement before intraperitoneal injection with PcpA- or PhtD-specific monoclonal antibodies (mAbs). The mice were then challenged 1 h later with a lethal dose of *Spn* serotype 3 strains A66.1 or WU2, injected intravenously. Control mice challenged with *Spn* alone died within 2 days, whereas mice injected with PcpA- or PhtD-specific mAbs survived for at least 10 days (Fig. 1). However, all mice injected with cobra venom factor to deplete complement died before day 10 despite the presence of PcpA- or PhtD-specific mAbs. In separate experiments, mice treated with cobra venom factor alone survived for the entire surveillance period (10 days) (Supplementary Table 1).

**Figure 1.**
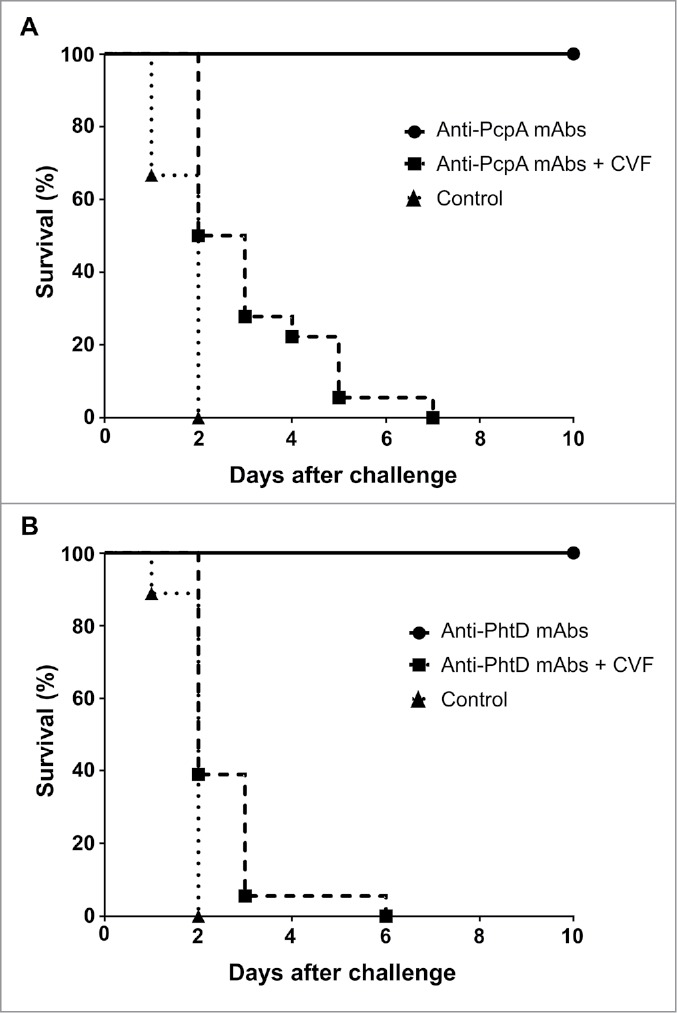
Complement depletion eliminates protection by PcpA- and PhtD-specific antibodies. Six- to eight-week-old female CBA/N mice (Jackson Laboratories, bred at Sanofi) received an intraperitoneal injection of a pool of two anti-PcpA mAbs (clones A-2B3.1.5 [IgG1] and A-1-12.2.2 [IgG2a]) at 10 µg per dose each (A) or a pool of three anti-PhtD mAbs (clones D8H6.12.3 [IgG2a], D-1B12.13 [IgG2b] and D-4D5.6 [IgG2b]) at 20 µg per dose each (B). Control animals received 60 µg of irrelevant mAbs. All mAbs were obtained from Harlan. Mice were challenged 1 h later with single 200-µl intravenous injections of 50 colony-forming units of *Spn* serotype 3 strain A66.1 (A) or 600 colony-forming units of *Spn* serotype 3 strain WU2 (B), which expresses higher surface levels of PhtD (our unpublished observations). *Spn* serotypes were cultured as previously described.^16^ Complement was depleted in the indicated mice by intraperitoneal injection of 10 international units/kg of cobra venom factor (CVF; Quidel, #A600) before and 3 and 6 days after challenge with *Spn*. Survival was followed for 10 days. Data in A and B depict one of two determinations with similar results (n = 9 per group). All animal experiments were conducted with the approval of institutional and national animal care committees.

**Table 1.** Effects of neutrophil depletion, splenectomy, and macrophage depletion on protection mediated by PcpA- and PhtD-specific antibodies.

						Surviving, n (%)		
*Spn* strain	mAb	Splene- ctomy^a^	Neutro-phils depleted^b^	Macro- phages depleted^c^	No. Mice	Day 1	Day 2	Day 10
A66.1	Anti-PcpA	−	−	−	8	8 (100.0)	8 (100.0)	8 (100.0)
	Anti-PcpA	−	+	−	16	16 (100.0)	16 (100.0)	15 (93.7)
	Anti-PcpA	+	−	−	16	15 (93.7)	15 (93.7)	13 (81.2)
	Anti-PcpA	+	+	−	16	16 (100.0)	16 (100.0)	16 (100.0)
	Irrelevant	−	−	−	15	15 (100.0)	8 (53.3)	1 (6.7)
A66.1	Anti-PcpA	−	−	−	8	8 (100.0)	8 (100.0)	8 (100.0)
	Anti-PcpA	−	−	+	16	2 (12.5)	0 (0.0)	0 (0.0)
	Irrelevant	−	−	−	15	15 (100.0)	8 (53.3)	1 (6.7)
WU2	Anti-PhtD	−	−	−	8	8 (100.0)	8 (100.0)	8 (100.0)
	Anti-PhtD	−	+	−	8	8 (100.0)	8 (100.0)	8 (100.0)
	Anti-PhtD	+	−	−	8	8 (100.0)	8 (100.0)	8 (100.0)
	Anti-PhtD	+	+	−	8	8 (100.0)	8 (100.0)	8 (100.0)
	Irrelevant	−	−	−	8	8 (100.0)	2 (25.0)	0 (0.0)
WU2	Anti-PhtD	−	−	−	16	16 (100.0)	16 (100.0)	16 (100.0)
	Anti-PhtD	−	−	+	16	16 (100.0)	0 (0.0)	0 (0.0)
	Irrelevant	−	−	−	16	16 (100.0)	0 (0.0)	0 (0.0)

As described in Fig. 1, CBA/N mice received an intraperitoneal injection of anti-PcpA, anti-PhtD, or irrelevant mAbs. Mice were challenged 1 h later with single intravenous injections of a lethal dose of *Spn* A66.1 or WU2 strains. Survival was followed for 10 days.

a Splenectomy was performed on anesthetized mice 2 weeks before passive immunization and lethal challenge with the indicated *Spn* strain (D0). Control mice were sham-operated. Before and 1 day after surgery, mice were subcutaneously administered 0.1 mg/kg buprenorphine.

b 1 day before and 3 and 7 days after bacterial challenge, mice were treated by intraperitoneal injection with PBS containing 600 μg of anti-Ly6G mAb (clone 1A8; BioXCell, #BE0075) to deplete neutrophils as described previously.^39^ Control mice received PBS alone. Depletion of blood neutrophils by at least 90% was confirmed by flow cytometry (data not shown).

c 3 days before and 1 day after bacterial challenge, mice were injected intravenously with 1 mg clodronate liposomes (from Dr N. Van Rooijen, clodronateliposome.org, #283539) in PBS to deplete macrophages as previously described.^40^ Control mice received PBS alone.

In agreement with this requirement for complement in the passive protection model, complement deposition on *Spn* was promoted by anti-PcpA or anti-PhtD mAbs, hyperimmune sera from rabbits immunized with PcpA- or PhtD-monovalent vaccines, and post-immune sera from human subjects vaccinated with a PcpA- and PhtD-bivalent PPrV^14^ (Fig. 2). Therefore, and because *Spn* is resistant to the complement membrane attack complex,^23^ anti-PcpA and anti-PhtD antibodies likely promote clearance by enhancing complement-mediated phagocytosis.

**Figure 2.**
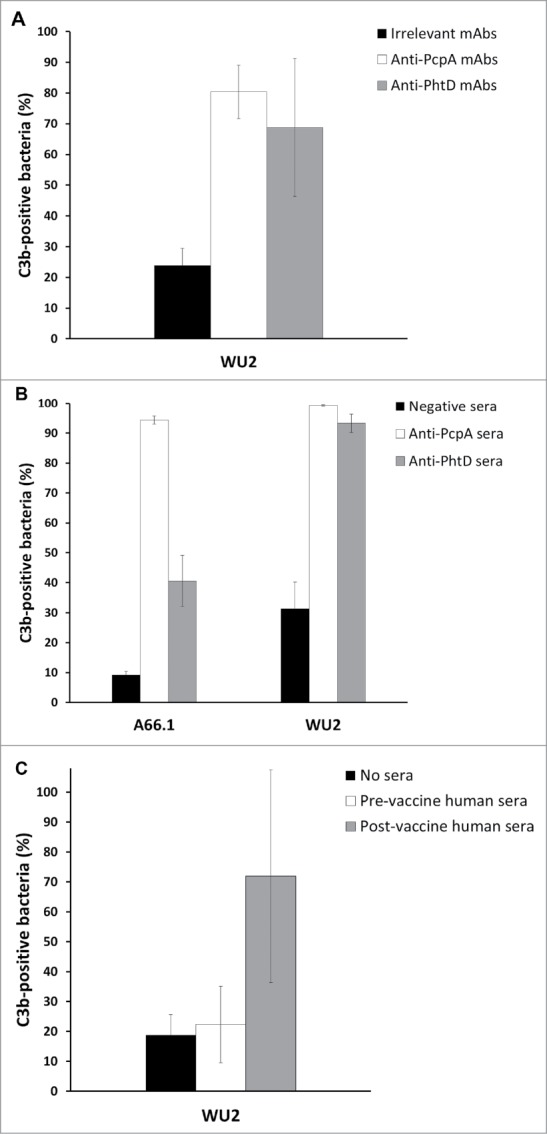
PcpA- and PhtD-specific mAbs and sera promote complement C3 deposition on *Spn. Spn* strains WU2 or A66.1 (1.3 × 10^6^ colony-forming units) in 20 μl assay buffer (phosphate-buffered saline + 1% bovine serum albumin) were incubated for 30 min at 37°C with an equal volume of pooled anti-PcpA or anti-PhtD mAbs (see Figure 1 legend; 50 µg/ml final concentration of each mAb) (A), hyperimmune sera from rabbits vaccinated with monovalent PcpA or PhtD vaccines formulated with a proprietary squalene-based TLR4 adjuvant (1:40 final concentration; Sanofi, Montpellier) (B), or pooled pre- or post-immune sera from human subjects vaccinated with a bivalent PcpA-PhtD PPrV in a clinical trial^14^ (1:320 final concentration) (C). To deplete complement, all sera were heated before mixing with *Spn*. Opsonized bacteria were then washed twice in assay buffer and incubated with 13% (A and B) or 9% (C) baby rabbit complement (in-house preparation) for 90 min at 37°C. Next, bacteria were incubated for 30 min at 37°C with 1:100 fluorescein isothiocyanate-conjugated goat anti-rabbit C3 antibody (MP Biomedical, #0855654), and the percentage of antibody-bound bacteria was determined using an Accuri C6 flow cytometer (Becton Dickinson) and analyzed using CSampler software (Becton Dickinson). Bars indicate means and error bars indicate standard deviations. In A, results depict the means of five determinations for anti-PcpA and anti-PhtD mAbs and two determinations for irrelevant mAbs; in B, of three determinations; and in C, of two determinations. All flow cytometry evaluations were based on ≥ 20,000 gated events.

Because neutrophils and macrophages are the major cell types mediating phagocytosis of *Spn,*^11,18,24^ we next examined which of these cell types are required for passive protection by anti-PcpA and anti-PhtD mAbs. Depleting neutrophils using anti-Ly6G mAbs, splenectomy, or a combination of both did not affect passive protection by anti-PcpA and anti-PhtD mAbs (Table 1). However, passive protection was eliminated when macrophages were depleted using clodronate liposomes, so that all mice died within 2 days. Thus, macrophages, but not neutrophils, were required for anti-PcpA and anti-PhtD mAbs to passively protect mice against *Spn* in our model.

Mouse models of *Spn* infection, such as the CBA/N model, are routinely used to quantify the protection by candidate PPrVs in clinical trials.^22^ However, the ethical considerations, technical difficulties, and time required for this model have motivated research into *in vitro* assays.^9,20,25^ We therefore applied our findings to help develop an *in vitro* opsonophagocytosis assay (Fig. 3). Anti-PcpA and anti-PhtD rabbit sera increased the phagocytosis of fluorescently labeled *Spn* by human granulocytes in the presence of complement. However, we did not observe phagocytosis of *Spn* by freshly isolated neutrophils from CBA/N mice, the J774A.1 mouse macrophage cell line, or whole blood cells from CBA/N mice (data not shown).

**Figure 3.**
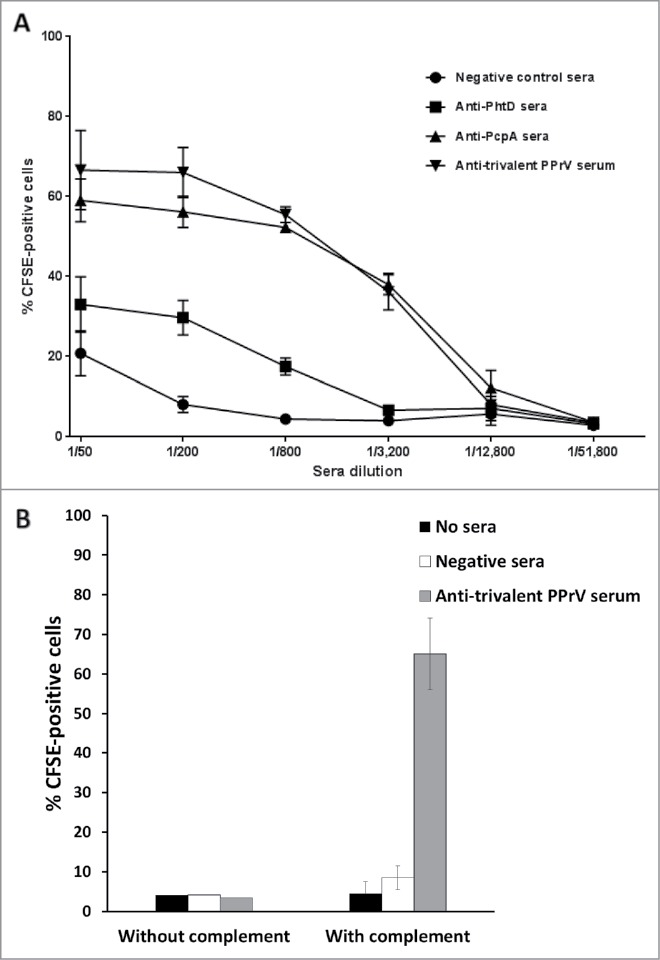
PcpA- and PhtD-specific rabbit sera promote *Spn* phagocytosis by human granulocytes. *Spn* WU2 fluorescently labeled with 6-carboxyfluorescein succinimidyl ester (CFSE; Thermofisher, #C1311) (1 × 10^6^ colony-forming units in 50 µl) were mixed with 50 µl of a 1:1:1 mixture of assay buffer (RPMI1640 + 5% fetal calf serum), 2% baby rabbit sera (in-house preparation) as the source of complement, and dilutions of heat-inactivated hyperimmune sera from rabbits immunized with monovalent PcpA or PhtD vaccines (A) or 1:200 hyperimmune sera (final concentration) from rabbits immunized with a trivalent PcpA-PhtD-PlyD1 vaccine or adjuvant alone (negative sera) (B). After 30 min at 37°C, washed human granulocytes (1 × 10^5^ cells in 200 µl) were added. After 30 min at 37°C, percentages of CFSE-positive phagocytic cells were determined using an Accuri C6 flow cytometer (Becton Dickinson) and CSampler software (Becton Dickinson). Mean percentages of CFSE-positive cells are shown with error bars indicating the standard deviation. All flow cytometry evaluations were based on ≥ 10,000 gated events. Results depict the means of (A) three determinations, and (B) one determination (without complement) or four determinations (with complement).

Together, our results suggest that trivalent PPrVs protect against *Spn* not only by inducing pneumolysin-neutralizing antibodies^17^ but also by promoting complement-dependent opsonophagocytosis by macrophages. This also suggests that the PhtD and PcpA antigens in the trivalent PPrV can induce antibodies that protect by this mechanism. Complement is similarly required for human anti-pneumococcal IgG to protect against infection and bacteremia-associated complications^26^ and is further required for maximal induction of phagocytosis by antibodies against pneumococcal surface protein A (PspA).^19,20,25,27–30^

Vaccination with a trivalent PPrV has been suggested to enhance early clearance of *Spn* from the lungs of mice by increasing phagocytosis by neutrophils.^18^ Similarly, transfer of pneumococcus-immunized serum increases *Spn* uptake by mature splenic neutrophils, and this uptake is complement-dependent.^31^ Consistent with these findings, we showed that human granulocytes phagocytozed *Spn* in the presence of complement and anti-PcpA or anti-PhtD antibodies. However, neutrophils were not needed for anti-PcpA and anti-PhtD antibodies to passively protect CBA/N mice against an intravenous lethal challenge with *Spn*. This discrepancy might be explained by the partially impaired neutrophil maturation and function in this mouse strain.^32^ Indeed, a recent study in C57BL/6 mice showed mature splenic neutrophils are integral for *Spn* clearance.^31^ In our mouse model, macrophages and complement were indispensable for protection by anti-PcpA and anti-PhtD antibodies. Interestingly, macrophages, but not neutrophils, are similarly required for *Spn* clearance by mAbs against *Spn* polysaccharide capsule antigens.^33^ Further study in other *Spn* infection models will be needed to make a more definitive conclusion about the role of neutrophils in passive protection by anti-PcpA and anti-PhtD mAbs.

Splenectomized patients are highly susceptible to infection by new *Spn* strains for which they do not have pre-existing immunity.^34^ This is not only because they have impaired IgM antibody responses to polysaccharide antigen,^34^ but also because splenic macrophages and neutrophils are likely to control the early stages of *Spn* infection before sufficient antibody levels are raised.^31^ In our study, splenectomy did not affect passive protection by anti-PcpA and anti-PhtD mAbs, as previously observed for passive protection by anti-PspA antibodies.^35^ This suggests macrophages outside of the spleen eliminated opsonized *Spn*, likely those in the liver.^36–38^ Liver-resident Kupffer cells, for example, clear C3-opsonized bacteria in the circulation via their CRIg receptors.^38^

In addition to clarifying the mechanism of protection by the trivalent PPrV, our results indicate some options for developing assays to rapidly assess functional antibody responses to PPrVs in clinical trials. For example, antibody responses could be measured by complement deposition assays or by a modified opsonophagocytosis assay similar to that proposed to study anti-PspA antibodies.^9,25^

In conclusion, our study suggests that anti-PhtD and anti-PcpA antibodies induced by PPrVs protect against *Spn* by a complement- and macrophage-dependent opsonophagocytosis.

Note: The findings presented in this manuscript were derived from repeat experiments and are supported by clear-cut differences between compared experimental conditions (such as 0% versus 100% survival), which indicates the results' practical significance; hence, no analysis to show statistical significance was performed.

## Supplementary Material

KHVI_A_1403698_Supplemental.docx
