# Supplementary material for: Antibodies to PcpA and PhtD protect mice against Streptococcus pneumoniae by a macrophage- and complement-dependent mechanism
Source: Hum Vaccin Immunother. 2017 Dec 14;14(2):489–94. doi: 10.1080/21645515.2017.1403698 (PMC5806646; doi:10.1080/21645515.2017.1403698)
Supplement: KHVI_A_1403698_Supplemental.docx [file khvi-14-02-1403698-s001.docx]

# Supplemental Material for Article: Antibodies to PcpA and PhtD protect mice against *Streptococcus pneumoniae* by a macrophage- and complement-dependent mechanism

**Supplementary Table 1. Effects of complement depletion by cobra venom factor on mouse survival**

|  |  | **Surviving, n (%)** | | | |
| --- | --- | --- | --- | --- | --- |
| ***Experiment*** | **No. Mice** | **Day 1** | **Day 2** | **Day 5** | **Day 10** |
| Experiment 1 | 5 | 5 (100.0) | 5 (100.0) | 5 (100.0) | 5 (100.0) |
| Experiment 2 | 5 | 5 (100.0) | 5 (100.0) | 5 (100.0) | 5 (100.0) |

Six- to eight-week-old female CBA/N mice (Jackson Laboratories, bred at Sanofi) received an intraperitoneal injection of 10 international units/kg of cobra venom factor (CVF; Quidel, #A600) on days 1, 3, and 6. Survival was followed for 10 days.
